# Photothermal Effect: The Amygdaloidal Nano-Structure Based on Bi_2_S_3_ for the Enhanced Degradation of Rhodamine B Under Irradiation by NIR

**DOI:** 10.3389/fchem.2021.680632

**Published:** 2021-05-28

**Authors:** Xiuzhao Yin, Yunyu Zhang, Fujin Ai

**Affiliations:** ^1^ College of Health Science and Environmental Engineering, Shenzhen Technology University, Shenzhen, China; ^2^ Chemical Engineering Institute, Xiamen University, Xiamen, China

**Keywords:** structure, nanophotocatalyst, photothermal effect, Bi_2_S_3_, degradation

## Abstract

In recent years the photothermal effect, an auxiliary strategy for increasing the degradation rate of pollutants under irradiation by near-infrared (NIR), has become a research focus. In this study a novel amygdaloidal nanophotocatalyst, Bi_2_S_3_, was synthesized by a traditional approach using a hydrothermal process, in which Bi_2_S_3_ nanostructures were spread out like a peacock’s tail. The produced Bi_2_S_3_ photocatalyst exhibited excellent performance in the rapid degradation of Rhodamine B (RB). This proved that the photothermal effect is mainly responsible for the rapid degradation of RB under NIR laser irradiation. Moreover, it was found that the photothermal effect could not degrade the products with NIR radiation in darkness. However, with the support of visible radiation, the photothermal effect of the Bi_2_S_3_ photocatalyst enhanced degradation of RB (degradation rate 90% under 1 h). This novel structure exhibited a potential ability for degrading pollution in industry or agriculture.

## Introduction

In recent years nanostructured photocatalysts have received much attention, owing to their outstanding performance in degrading dangerous organic pollutants(Hot et al., 2019; Ajibade and Mphahlele, 2021; Foo et al., 2021; Xu et al., 2021; Zhou et al., 2021). This unique nanostructure has good properties, which have been used in the degradation of toxic chemicals or dyes by adsorption, biological degradation, chlorination, and ozonation from traditional water (Hailing and Sang-Bing, 2017; Fessi et al., 2020; Fujin et al., 2021). Among nanostructured photocatalysts, the semiconductor is discrete, with a forbidden band between the valence band (VB) and the conduction band (CB). When energy is higher than the semiconductor absorption threshold of light semiconductor, semiconductor material carrier separation and valence electrons interband transition occur, producing light electron and hole (Zhang et al., 2011; Xiaosheng et al., 2012; Ju et al., 2014; Huang et al., 2016), and then holes and electronic or molecules and ions, forming free radicals that are reductive or oxidizing active, which can degrade macromolecular organic matter, carbon dioxide, water, or other small molecule organic matter (Hu et al., 2014; Wang et al., 2016). During the reaction process, the photocatalyst itself does not change. Valence band holes show strong oxidation ability and conducting electrons act as a reducing agent (Ohbuchi, 2003).

The two semiconductor heterojunctions can be classified into different categories according to the band positions that comprise them. Among them, Z-heterojunction photocatalytic materials have attracted attention, because the Z system retains not only the optical cavity with strong oxidation ability but also the photoelectron with strong reducing ability (Luo et al., 2016; Meng and Zhang, 2016; Sun et al., 2017a; b et al., 2017; Sun et al., 2017b; Zhu et al., 2017). Lin synthesized an Ag/Ag_3_PO_4_/Bi_2_Mo_6_ by *in situ* precipitation method (Xue et al., 2016), which is a highly efficient optical drive Z attributing to effectively separate the light electrons and holes.

The strength of photocatalytic performance mainly depends on two aspects. The first is the light absorption range of the semiconductor. The larger the light absorption range, the higher the utilization efficiency of sunlight, and vice versa. The other is the recombination rate of photogenerated electrons and partly hollow. The higher the recombination efficiency, the lower the quantum efficiency. In fact, since the photocatalytic properties of semiconductor materials were found, the study of the semiconductor photocatalyst modification began, whose goals include inhibition of the carrier compound, expanding the scope of the absorption wavelength of light, enhancing the stability of photocatalytic materials, and improving product yield, etc. (Hailing and Sang-Bing, 2017; Ma et al., 2018; Zhang et al., 2019), which are also the main strengths of the semiconductor photocatalyst performance index.

Many Bi group compounds have been reported, including Bi_2_O_3_, Bi_2_WO_6_, Bi_2_Ti_2_O_7_, Bi_2_S_3,_ and BiOCl (Zhang et al., 2010b; Fu et al., 2012; Yu et al., 2016). These semiconductor photocatalysts can be divided into general containing bismuth oxide bismuth (Chen et al., 2014; Jiang et al., 2015; Liang et al., 2016; Zhang et al., 2016; Sumathi et al., 2019), dual metal oxides, and halogen containing bismuth oxide (Walsh et al., 2010; Hong et al., 2011; b et al., 2021), most of which is active in the visible light region. The band gap is less than 3.0 eV. Bi_2_S_3_, BiOI (Zhang et al., 2006; Zhang et al., 2009; Cheng et al., 2010; Jiang et al., 2011), and a band gap of less than 2.0 eV show that they have the ability to absorb wavelengths longer than visible light. The photocatalytic activity of semiconductors is not only influenced by the band gap, it is also influenced by structure and morphology. Bi group compound synthesis and light catalytic techniques have been developed more recently.

Bi_2_S_3_, as a semiconductor photocatalyst with a narrow band gap (1.3–1.7 eV) and high visible light availability, has attracted extensive attention from researchers (Chen et al., 2013). The single component Bi_2_S_3_ photocatalyst has the disadvantages of severe photocorrosion and high charge recombination rate, which greatly limit the photocatalytic activity of visible light. Hu (Hu et al., 2013) and colleagues prepared a nano-Bi_2_S_3_ with an excellent performance by hydrothermal method, using template sodium dodecylbenzene sulfonate, bismuth nitrate, and N, N-dimethyldithiocarbamate dimethylamine salt as raw materials. Bi_2_S_3_ composite photocatalytic material has high visible light utilization, and effectively inhibits the electron-hole composite, improving photocatalytic activity, and indicating varied application prospects in the field of photocatalytic research.

The NIR region (700–1400 nm) is considered a biological window. The over-layer of NIR translates light energy into partially hot, which is rapidly applied in over-temperature treatment to kill a harmful organization or cells (Lee et al., 2015; Li et al., 2015; Liu et al., 2015; Zhang et al., 2015; Liu et al., 2019). The photothermal effect could also play a crucial role in the enhanced degradation of pollution. Studies have proven that Bi_2_S_3_ based nanomaterials have excellent photothermal effects when exposed to NIR (Song et al., 2018; Huang et al., 2019; Mo et al., 2020).

In the present study, a novel amygdaloidal nanophotocatalyst, Bi_2_S_3_, was synthesized by a traditional method by hydrothermal means, in which a Bi_2_S_3_ nanostructure was spread out like a peacock’s tail. The produced Bi_2_S_3_ photocatalyst had good ability in the rapid degradation of RB in industry. It proved that the photothermal effect is mainly responsible for the rapid degradation of RB under NIR laser irradiation. Moreover, it was found that the photothermal effect could not degrade products with NIR radiation in the dark. With the support of visible radiation, the photothermal effect of the Bi_2_S_3_ photocatalyst enhanced the degradation of RB (degradation rate 90% under 1 h).

## Experiments and Reagents

### Materials and Reagents

Ethylene glycol [(CH_2_OH)_2_], Polyvinylpyrrolidone (PVP) (wt = 13,000), N, N-Dimethylformamide (DMF), Ammonium thiomolybdate [(NH_4_)_2_MoS_4_] and Bismuth nitrate pentahydrate [Bi(NO_3_)_3_·5H_2_O] were purchased from Aldrich and used without any purification.

### Synthesis of Bi_2_S_3_ Nanoparticles

1 g PVP was dissolved in 20 ml DMF until all solids disappeared, then 0.15 g (NH_4_)_2_MoS_4_ and 0.3 g Bi(NO_3_)_3_·5H_2_O were added under vigorous stirring for 30 min, 40 ml (CH_2_OH)_2_ was added to the solution under vigorous stirring for 30 min, The mixture was heated to 180°C and maintained for 6 h. Subsequently, the solid black Bi_2_S_3_ nanocrystals were collected by centrifugation and washed with distilled water and ethanol three times. Finally, it was placed in a vacuum drying chamber for 6 h.

### Characterization

Powder X-ray diffraction (XRD) for structure characterization was performed on a D/max-2550 PC X-ray diffractometer (Rigaku, Japan). Scanning electron microscopy (SEM) was conducted on a JEM-2100F electron microscope at an acceleration voltage of 200 kV (JEOL, Japan). The UV-vis diffuse reflectance and absorption spectra were obtained from Lambda 35 spectrophotometer (PerkinElmer) and U-3100 spectrophotometer (Hitachi), respectively. The X-ray photoelectron spectra (XPS) were taken on a VG ESCALAB MK II electron spectrometer using Mg Kα (1,200 eV) as the excitation source. Thermal images were recorded using a FLIR T420 thermal camera.

### The Degradation of RB Activity

The photocatalytic performance of Bi_2_S_3_ nanomaterials was assessed by the degradation efficiency of RB under ultraviolet light (UVIR, 90 W), visible light (Philips, 40 and 90 W), and near-infrared laser irradiation (Armlaser Inc. United States, 2 W/cm^2^), 808 nm). In every comparison experiment, 10 mg of Bi_2_S_3_ nanomaterials was distributed in 100 ml of RB aqueous solution (10 mg L^−1^). The solution was stirred in the dark for 30 min to establish the adsorption/desorption equilibrium of RB molecules on the catalyst. Subsequently, the solution was added to a double-walled photocatalytic reactor with a water circulation system to keep the reaction mixture at 25°C. The suspension is then exposed to ultraviolet, visible, and near-infrared radiation respectively. In a given time interval, take out 5 ml of the suspension and centrifuge, and use a UV-vis spectrophotometer to analyze the concentration of RB by measuring the absorbance at 554 nm.

### The Photothermal Effect of Degradation

The photothermal effect of the samples was evaluated using an 808 nm NIR diode laser system (Armlaser Inc. United States) with an output power of 1 W cm^−2^. In each experiment, 1 ml of an aqueous dispersion of the sample was transferred into a 1 × 1 × 4 cm^3^ cuvette and illuminated with a NIR laser. Then the increase in temperature of the suspension was mediated by exposure to laser radiation and measured using a digital thermometer by immersing its thermocouple in the reaction mixture during the experiment.

### Extracellular·OH Detection

To assess the extracellular OH detection, 0.1 mm Bi_2_S_3_ nanomaterials were added in 3 ml of MB solution with pH values. Then, the mixture solution was stirred in darkness for 60 min to attain absorption-desorption equilibrium. After stirring for 0, 3, 6, and 9 min, then being centrifuged to remove Bi_2_S_3_ NPs. The degree of OH generation is reflected by the decrease of the absorbance at 664 nm.

## Results and Discussion

### Preparation and Characterization of the Bi_2_S_3_ NPs

**FIGURE 1:**
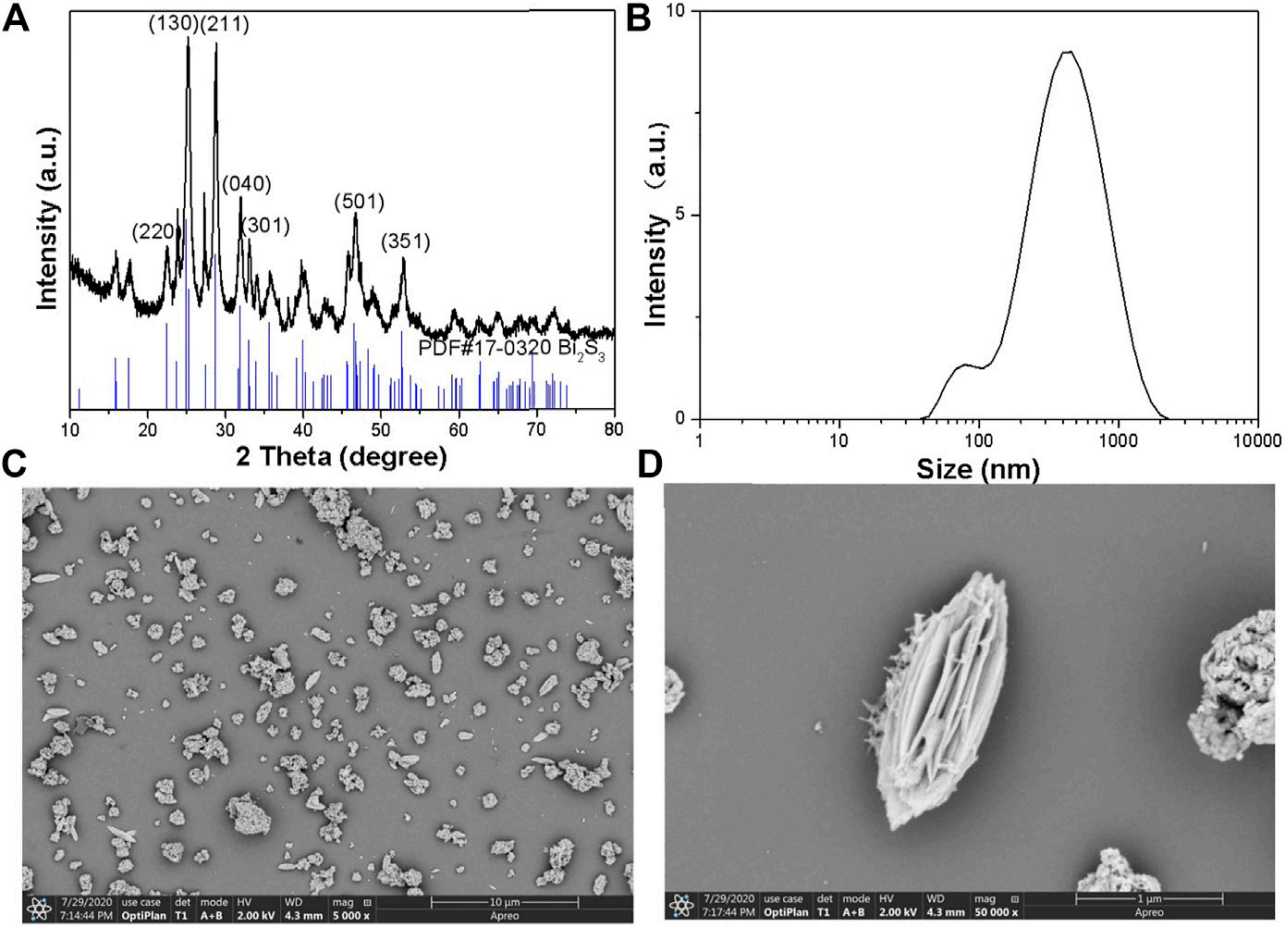
**(A)** Powder XRD patterns of the as-prepared nanocrystals and the standard Bi_2_S_3_ powders. **(B)** DLS of the Bi_2_S_3_ NPs. **(C)** The SEM images of the Bi_2_S_3_ nanocrystals. **(D)** Typical SEM images of the Bi_2_S_3_ nanocrystals.

The Bi_2_S_3_ was synthesized by a hydrothermal method in an ethylene glycol environment. Firstly, the PVP was dissolved in DMF, then (NH_4_)_2_MoS_4_ and Bi(NO_3_)_3_·5H_2_O was added respectively in DMF under vigorous stirring. Subsequently, ethylene glycol was added to the solution. The mixture was translated into the hydrothermal reactor. At last, dark black products were gathered by centrifugation. The average edge length and width of this nanoflower is 557 nm, which can be further demonstrated by the Dynamic Light Scattering (DLS) (Figure 1B). A similar experiment can be further demonstrated by the higher magnification scanning electron microscopy (SEM) image (Figure 1C). Figure 1D shows Bi_2_S_3_ in the broken part of the enlarged photo. Empty microspheres can be observed from the figure. Every Bi_2_S_3_ nanometer ball was a length of approximately 500 nm. The Bi_2_S_3_ nanorods cross assemble into each other in a disorderly fashion. This kind of hollow porous structure light catalysis is very favorable. Furthermore, selected area electron diffraction (SAED) on individual dumbbells indicated the single-crystalline nature of these NCs. The structure of as-synthesized NPs was further confirmed by X-ray diffraction (XRD) (Figure 1A). The pattern could be well indexed to the orthorhombic Bi_2_S_3_ phase (JCPDS no. 17–0320). The characteristic peaks of Bi_2_S_3_ samples are consistent with those reported in the literature. The diffraction peaks at two Theta 24.9°, 28.6°, 31.6°, 32.9°, 46.7°, and 52.6° correspond to the crystal planes of (130), (211), (040), (301), (501), and (351), respectively. The crystal cell is slightly expanded, and the peak shape of Bi_2_S_3_ is sharp. There is no other diffraction peak, indicating that the prepared orthogonality crystal phase Bi_2_S_3_ has high purity.

### Photothermal Effect of the Bi2S3 NPs

**FIGURE 2:**
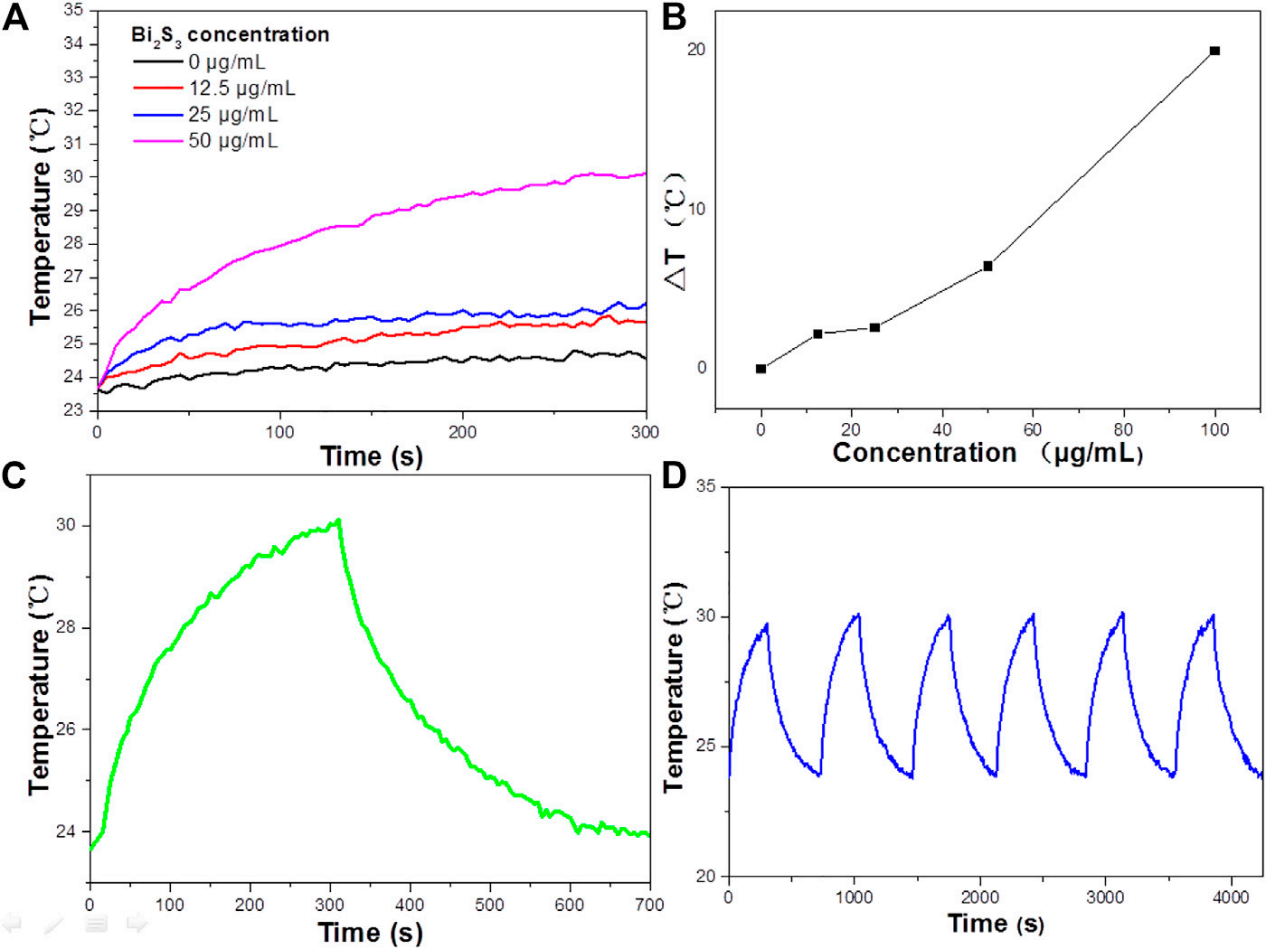
**(A)** The Absorbance spectra of RB standard solutions of different concentrations. **(B)** Absorbance-concentration fitting curve of RB solution. **(C)** UV—vis absorbance spectrum for the aqueous dispersion of the Bi_2_S_3_ NPs. **(D)** The reduced rate of RB on the surface of the Bi_2_S_3_ NPs under UV light, visible light, and NIR laser irradiation, all light power density is 1 w/cm^2^.

**FIGURE 3:**
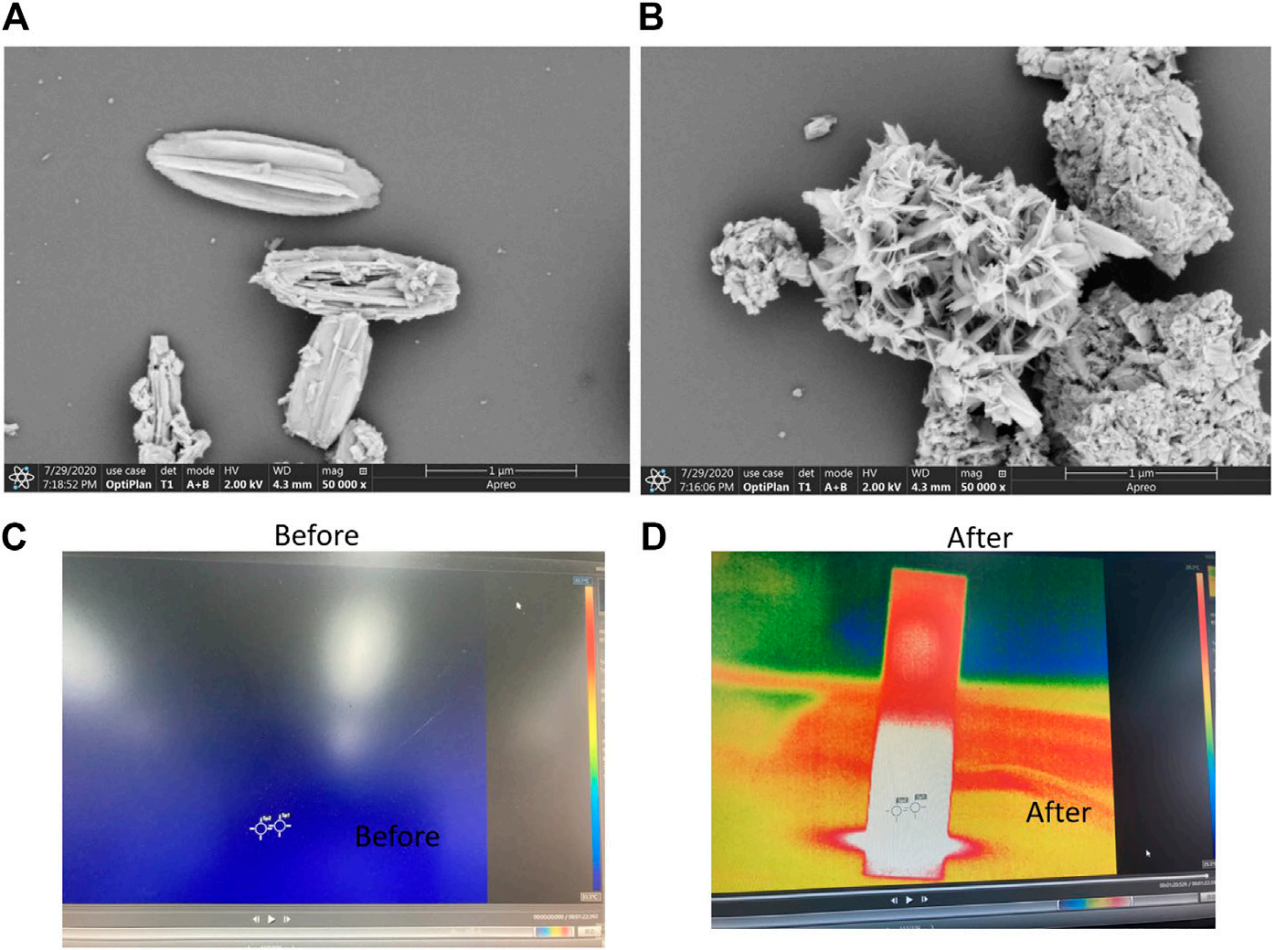
**(A)** Temperature elevation curves of aqueous dispersions containing the Bi_2_S_3_ NPs with different concentrations under laser irradiation. **(B)** Plot of temperature change (ΔT) vs. concentration of the Bi_2_S_3_ NPs. **(C)** Aqueous solution temperature change curve of the Bi_2_S_3_ NPs at 50 μg/ml under 808 nm NIR(1 W cm^−2^, 5 min) and the temperature decreasing process. **(D)** Aqueous solution temperature change curve of the Bi_2_S_3_ NPs under laser on/off cycles in solution.

**FIGURE 4:**
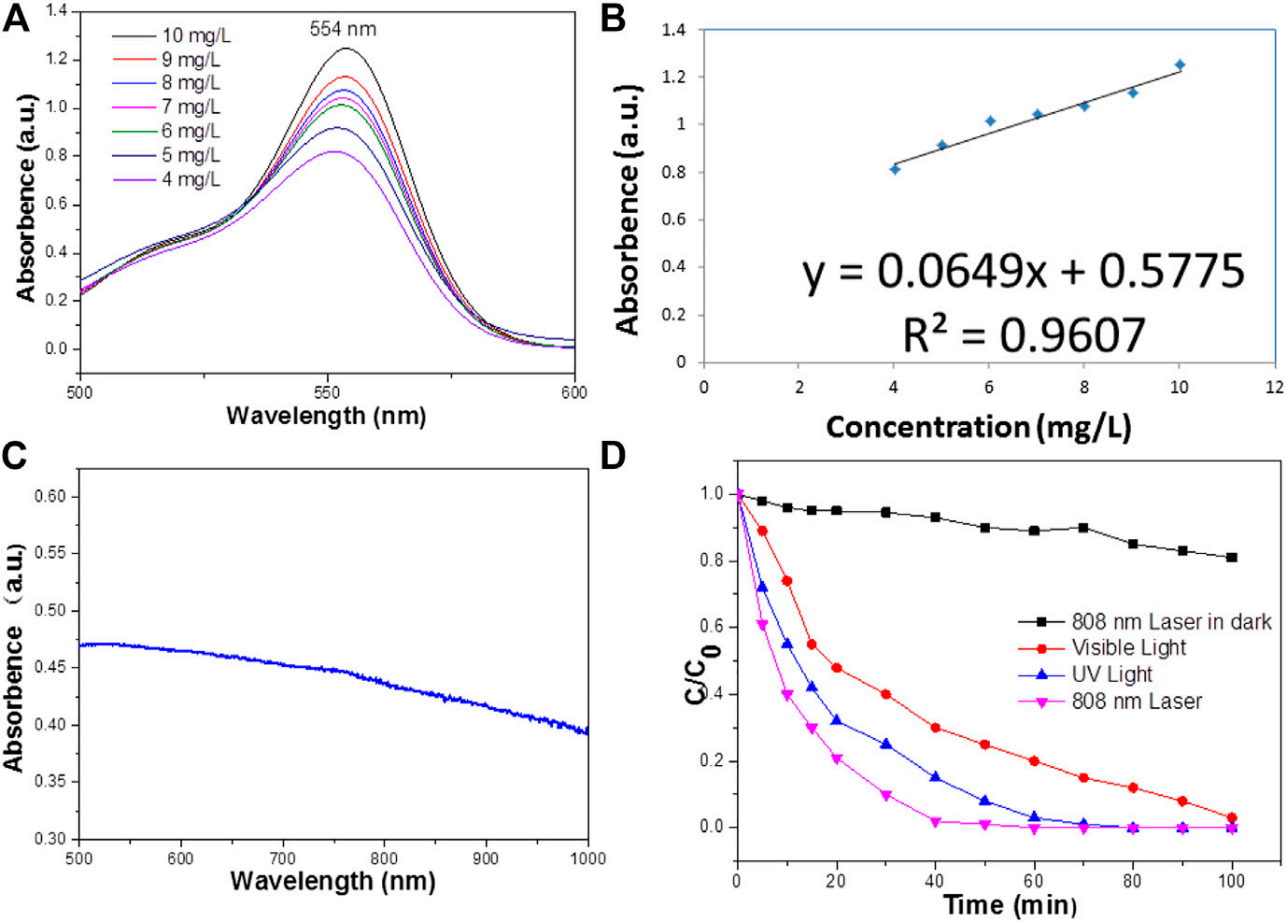
**(A)** SEM images of the Bi_2_S_3_ nanocrystals in front of the laser irradiation. **(B)** Some SEM images of the Bi_2_S_3_ nanocrystals after the laser irradiation. **(C)** Temperature condition of water and aqueous dispersions containing the Bi_2_S_3_ NPs at 50 μg/ml upon 808 nm in front of laser irradiation (1 W cm^−2^, 5 min). **(D)** Temperature condition of water and aqueous dispersions containing the Bi_2_S_3_ NPs after the laser irradiation.

Experimental data indicated that Bi_2_S_3_ has good NIR absorption (Figure 2C) and can be used as a Photocatalytic reagent, so we evaluated the photothermal effect of Bi_2_S_3_ NPs. The photothermal property of the Bi_2_S_3_ NPs, aqueous dispersions with various concentrations (0, 12.5, 25, and 50 μg/ml) were excited by an 808 nm laser. Figure 3A showed the temperature change curves with different Bi_2_S_3_ NPs concentrations. A concentration of 12.5 μg/ml showed a temperature increase of 2°C when exposed to light. With the concentration of the Bi_2_S_3_ NPs increased to 50 μg/ml, the temperature of the solution increased 8.1°C. While the temperature of pure water changed by 0.3°C (Figure 3B). Furthermore, the temperature increased by 20°C when the NPs concentration was 100 μg/ml (Figure 3B). As indicated by the temperature change curves from Figure 4C, the photothermal conversion performances of these Bi_2_S_3_ NPs were tested through exposure to irradiation with an 808 nm laser for 5 min at a power intensity of 1 W cm^−2^. The efficiency of light-to-heat was counted as 24.7% by using an established method (Gao et al., 2018; Tsai and Ma, 2018). The temperature of the Bi_2_S_3_ NPs solution was added from 23.2 to 30.3°C at the same time. In addition, the laser on–off circulation tests demonstrate the stability of the Bi_2_S_3_ NPs under photoexcitation (Figure 3D). This indicated that Bi_2_S_3_ NPs have good photothermal stability.

As indicated in Figure 4A, the comparative analysis of the morphology of nanoparticles before and after illumination revealed that there was a fixed morphology before illumination. After illumination, the morphology was clustered together, but the layered structure still existed (Figure 4B). This may be because constant agitation causes the nanoparticles to come together, and heat promotes the cracking of the structure in the presence of light. However, the photothermal stability was still very good, which may be due to the light reflecting back and forth on the surface of the nanoparticles. Some images indicate that when Bi_2_S_3_ nanoparticles are excited by near-infrared light, the temperature increases over time (Figure 4C). This promotes the degradation of organic dyes.

### The Performance of Photocatalytic

According to Lambert–Beer law

$$\text{A }=\text{ lg}\left({\text{I}}_{\text{0}}/\text{I}\right)=\text{Ig }\left(1/\text{T}\right)\;=\text{ kcd}$$

According to the formula, there is a linear relationship between the absorbance of the solution and the concentration of the dye solution, which provides an operational method for measuring the concentration of the solution by the absorbance. Therefore, standard solutions with different concentrations of dyes need to be configured before the experiment.

After the absorbance is measured, the absorbance-concentration relationship curve of the standard solution is drawn and the linear relationship between absorbance and concentration is fitted according to the data. Using this linear relationship, the absorbance of the dye can be measured and then converted to the concentration. Then, the degradation rate calculation formula can be used to calculate the degradation efficiency D of the sample to the dye, as follows:

$$\text{D }=\;\left({\text{C}}_{\text{0}}-{\text{C}}_{\text{t}}\right)/{\text{C}}_{\text{0}}\text{ × }100\text{\%}$$

Among, C_0_ is the concentration of the dye solution at adsorption equilibrium, and C_t_ is the concentration of the solution at the time of light. At room temperature, in advance, a certain quality of RB was dissolved in deionized water and ultrasonic for 3–5 min until it is completely dissolved. The high concentration solution drainage was then transferred to a 1 L volumetric flask with a glass rod in the beaker with deionized water and glass rod cleaning three times before the lotion was transferred to a volumetric flask. A glue dropper was used to join the deionized water head volumetric flask scale line flush with the solution under the liquid surface and then the homogeneous solution was shaken.

To improve the accuracy of the standard curve, RB solutions of 50 mg L^−1^ were prepared as standard solutions according to the above methods to reduce the concentration error. They were then diluted into dilute solutions of 4–10 mg L^−1^ and measured for absorbance. The absorbance spectrum curves of the standard solution are shown in Figure 2A, which indicates that the characteristic absorption peaks of RB are located at 554 nm. This is due to different hair color groups corresponding to different dyes, so the corresponding absorption wave positions of the characteristic peaks are different. The absorbance values corresponding to the characteristic absorption peak positions of the above dyes with different concentrations are shown in Figure 2B.

Water at enhanced temperatures can promote a favorable condition for the cleavage of the heterolytic bonds of the functional groups such as –OH and C=O persisting over the surface of Bi_2_S_3_ NPs. Therefore, it is obvious that the characteristic band of RB located around 554 nm has completely disappeared, which suggests that the structural rupture of RB molecules and the subsequent degradation of them by the Bi_2_S_3_ nanocatalyst.

The degradation of RB in the presence of NIR laser irradiation was compared with the degradation rate measured in visible and UV radiation (Figure 2D). The results showed that when exposed to visible light, RB is completely degraded at 100 min, while it needs 60 min under NIR irradiation. By contrast, under ultraviolet radiation, the photodegradation rate of RB reached 80 min, and under near-infrared radiation, the photodegradation rate of RB was higher than that of visible light and ultraviolet radiation. The excellent performance of Bi_2_S_3_ NPs under near-infrared laser irradiation may be related to the photothermal effect in the photocatalysis process.

In addition, the reduced rate of RB under the NIR radiation in the dark is much less than that in NIR radiation, except that there are lights in the laboratory (Figure 2D). It can be seen that 808 nm Laser radiation plays an important part in enhancing the reduced rate of Bi_2_S_3_ NPs. The degradation of RB potentially only occurs under NIR radiation, and a small amount of ultraviolet and visible light is required. Therefore, NIR radiation cannot accelerate the generation of electrons in Bi_2_S_3_ NPs, and NIR cannot increase the generation of electrons in Bi_2_S_3_ NPs. However, a little heat from the combination of UV or visible light can significantly accelerate the degradation process under NIR, which may have responsibilities in promoting the degradation of RB. Photocatalysis is the separation and transfer of light-generated carriers, so the photothermal effect plays an important role in enhancing the degradation of RB. The photothermal effect causes the temperature to rise and promotes the migration of carriers in Bi_2_S_3_ NPs, which leads to the rapid photodegradation of RB.

**FIGURE 5:**
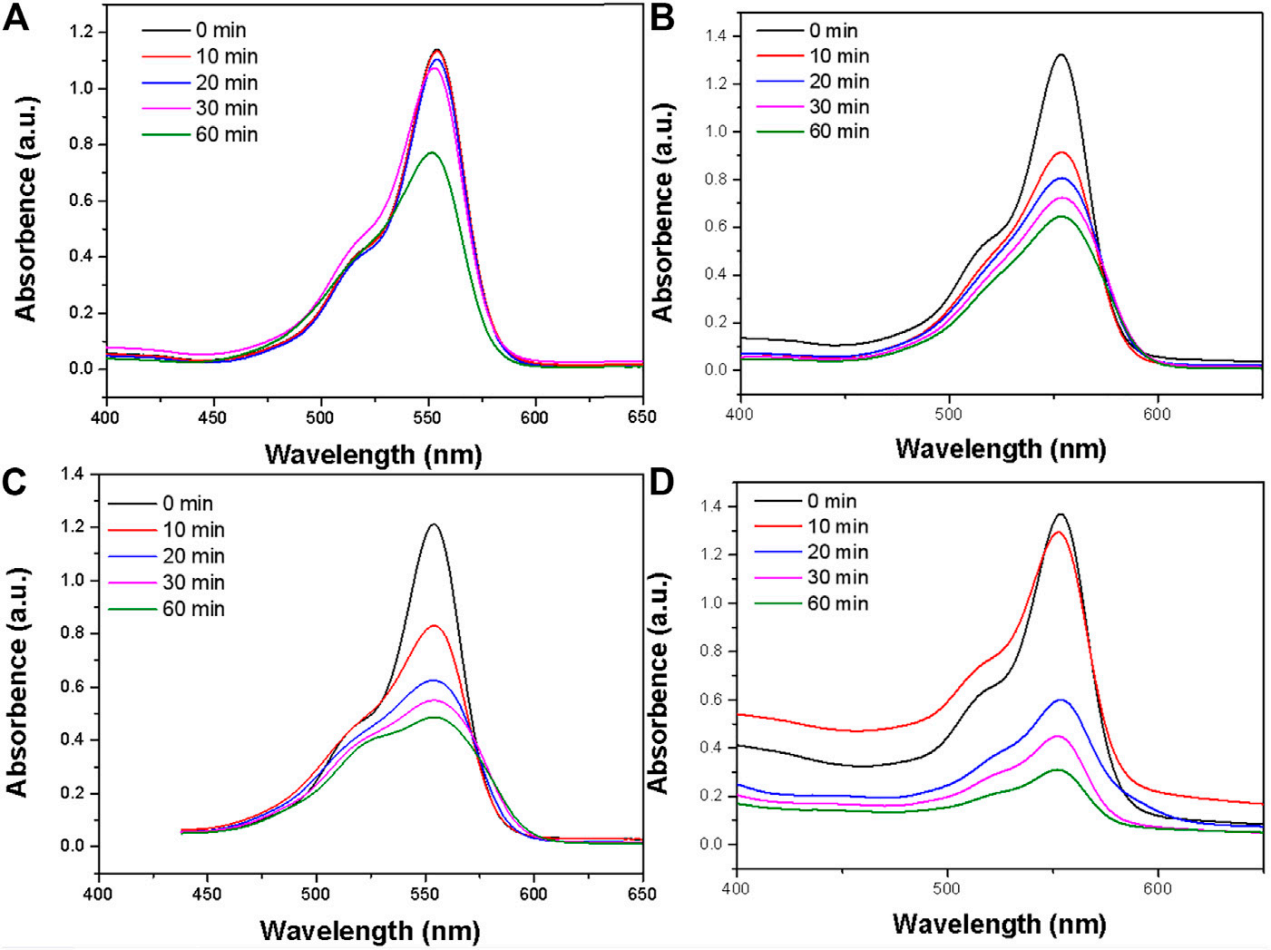
**(A)** The photodegradation profile of RB in the presence of the Bi_2_S_3_ nanocatalyst under pure heat experiment conditions. **(B)** The photodegradation profile of RB in the presence of the Bi_2_S_3_ nanocatalyst under commercial Bi_2_S_3_ nanocatalyst. **(C)** The photodegradation profile of RB in the presence of the Bi_2_S_3_ nanocatalyst under UV + NIR experiment conditions. **(D)** The photodegradation profile of RB in the presence of the Bi_2_S_3_ nanocatalyst under visible + NIR experiment conditions, the concentration of Bi_2_S_3_ nanocatalyst is 50 ppm in all instances.

The amygdaloidal nano-structure Bi_2_S_3_ photocatalyst exhibited better performance in the rapid degradation of RB than other photocatalysts, and enhanced heat could promote the rate of degradation. Pure heat can degrade organic dyes slowly (Figure 5A) and continued high temperatures may lead to the breakdown of molecular chains and break the links between molecules, thus degrading organic pollutants. Commercial Bi_2_S_3_ particles can also effectively degrade RB (Figure 5B), but compared with the special structure of the photosensitive catalyst, UV light, and 808 nm light simultaneously and irradiation of RB with the catalyst, the degradation rate can be rapid (Figure 5C). The same experimental data showed that visible light combined with near-infrared light at 808 nm accelerated the degradation of RB (Figure 5D). This apricot-like structure can decompose organic pollutant molecules under the stimulation of light, and heat promotes the degradation rate.

### The Mechanism of Enhanced Degradation

The degradation of RB by employing the Bi_2_S_3_ nanocatalyst could be ascribed to its high surface area under NIR laser irradiation loaded UV and visible light, which enhances the adsorption of RB molecules due to the p–p interaction between the aromatic ring of the RB molecules and Bi_2_S_3_ NPs, which leads to the noncovalent adsorption of dye molecules (Zhang et al., 2010a). Existing research shows that the transition metal sulfides have unique physical optoelectronic properties, and can be used as a novel and efficient catalytic agent. The valence band generally consists of S p3. Relative to the O 2p orbital energy level, it is more negative, and therefore, relative to the oxide of transition metal sulfides band gaps, meaning it can be smaller and is more likely to be sparked by the visible light. This means it has potential application prospects in the field of photocatalytic oxidation.

**FIGURE 6:**
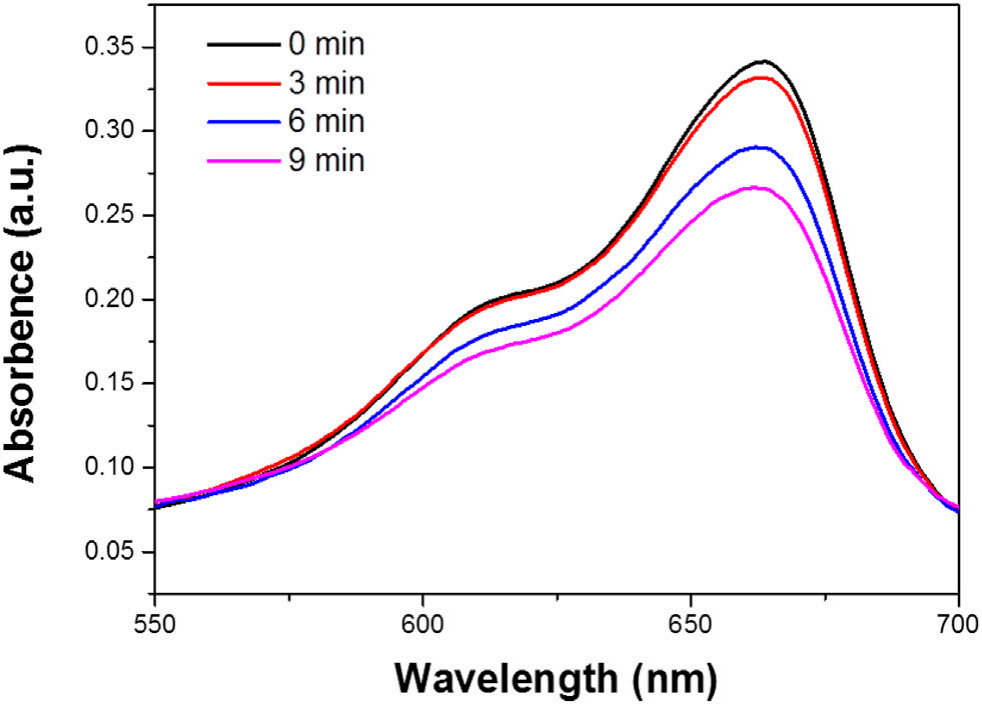
The mechanism for the degradation of RB in the presence of the Bi_2_S_3_ NPs under NIR laser.

**FIGURE 7:**
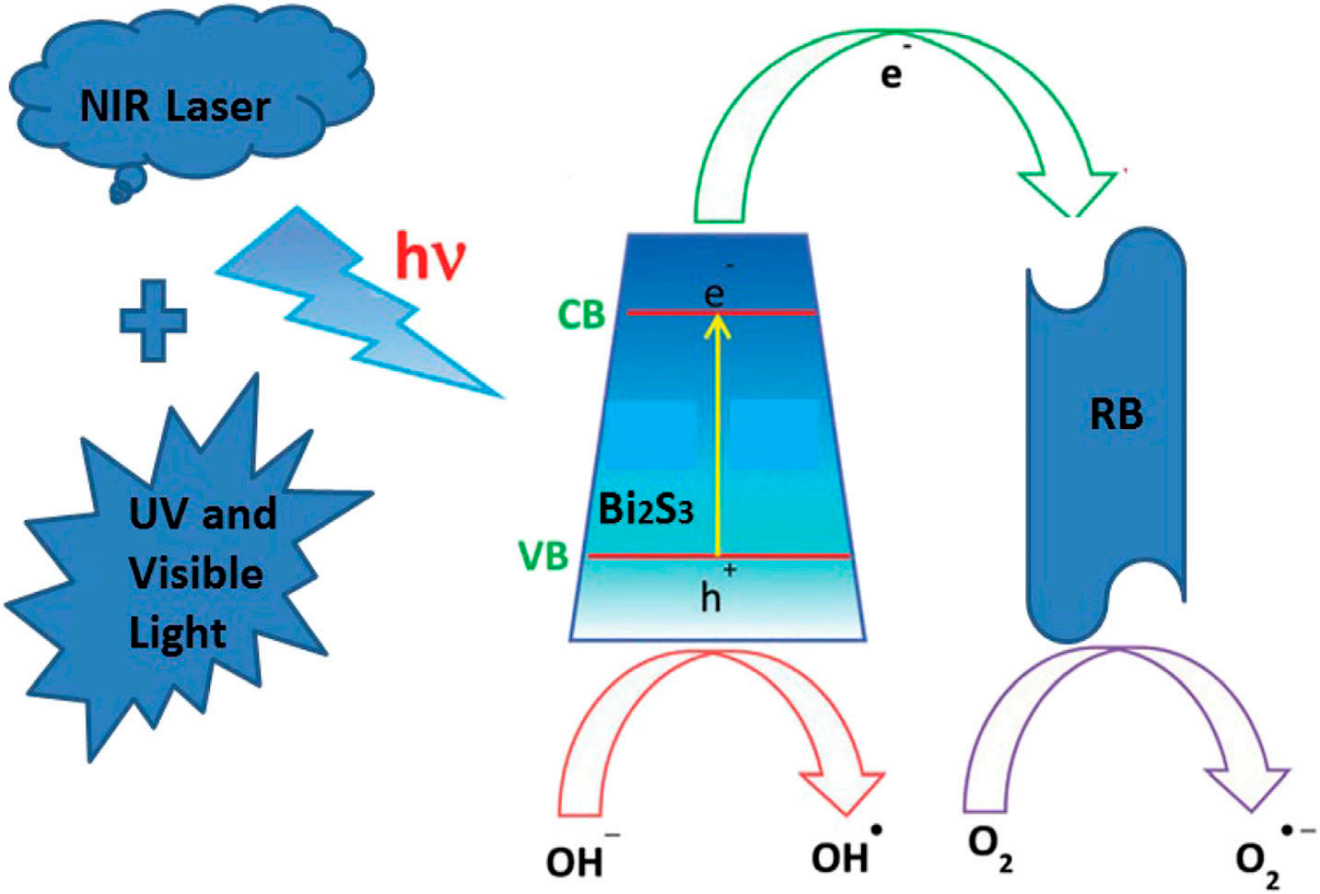
The degradation of MB in the Bi_2_S_3_ NPs in aqueous solution, the absorption value was measured with different treatment (0, 3, 6, and 9 min).

The mechanism underlying the photocatalytic activity of the Bi_2_S_3_ NPs in the degradation of RB under 808 nm Laser is shown in Figure 6. The Bi_2_S_3_ NPs nanocatalyst aqueous solution and RB are exposed to light to generate electron pairs (e) and holes (h+). The photothermal effect promotes the transfer of electrons from the valence band conduction band by leaving the valence band holes. This process significantly reduces the probability of light-excited electrons combining with holes in Bi_2_S_3_ NPs. At the same time, a large number of light-excited holes are retained and participate in the oxidation of RB, improving photocatalytic activity. More importantly, light-generated holes can react with adsorbed water to form hydroxyl radicals (OH) **(** Figure 7 **)**. The degree of OH generation is a response to the reduction of the absorbance strength at 664 nm, promoting the resolve of RB. Besides, O_2_ also leads to the generation of OH after protonation. OH and O_2_ are required for the resolve of RB under light irradiation (Liu et al., 2013).

## Conclusion

In the present study, an amygdaloidal Bi_2_S_3_ nanophotocatalyst was prepared by a facile hydrothermal process. Our findings revealed that layered nanostructures exhibit much higher photocatalytic activities under NIR loaded ultraviolet and visible light irradiation, which is attributed to increased optical absorption, decreased band width, and efficient classify carriers generated by light. This photothermal effect can promote the degradation of RB. However, under 808 nm Laser, the process, which photo produced, electrons could not happen.

## Data Availability

The original contributions presented in the study are included in the article/Supplementary Material, further inquiries can be directed to the corresponding author.
